# Hedgehog inhibition mediates radiation sensitivity in mouse xenograft models of human esophageal adenocarcinoma

**DOI:** 10.1371/journal.pone.0194809

**Published:** 2018-05-01

**Authors:** Jennifer Teichman, Lorin Dodbiba, Henry Thai, Andrew Fleet, Trevor Morey, Lucy Liu, Madison McGregor, Dangxiao Cheng, Zhuo Chen, Gail Darling, Yonathan Brhane, Yuyao Song, Osvaldo Espin-Garcia, Wei Xu, Hala Girgis, Joerg Schwock, Helen MacKay, Robert Bristow, Laurie Ailles, Geoffrey Liu

**Affiliations:** 1 Postgraduate Medical Education, University of Toronto, Toronto, Canada; 2 Department of Medical Biophysics, University of Toronto, Toronto, Canada; 3 Princess Margaret Cancer Centre, Toronto, Canada; 4 Department of Thoracic Surgery, University Health Network, Toronto, Canada; 5 Division of Biostatistics, Dalla Lana School of Public Health, Toronto, Canada; 6 Division of Epidemiology, Dalla Lana School of Public Health, Toronto, Canada; 7 Department of Laboratory Medicine and Pathobiology, Toronto, Canada; 8 Department of Medicine, Division of Medical Oncology, Sunnybrook Health Sciences Centre, Toronto, Canada; National Cancer Center, JAPAN

## Abstract

**Background:**

The Hedgehog (Hh) signaling pathway is active in esophageal adenocarcinoma (EAC). We used a patient-derived murine xenograft (PDX) model of EAC to evaluate tumour response to conventional treatment with radiation/chemoradiation with or without Hh inhibition. Our goal was to determine the potential radioresistance effects of Hh signaling and radiosensitization by Hh inhibitors.

**Methods:**

PDX models were treated with radiation, chemotherapy or combined chemoradiation. Tumour response was measured by growth delay. Hh transcript levels (qRT-PCR) were compared among frozen tumours from treated and control mice. 5E1, a monoclonal SHH antibody, or LDE225, a clinical SMO inhibitor (Novartis®) inhibited Hh signaling.

**Results:**

Precision irradiation significantly delayed xenograft tumour growth in all 7 PDX models. Combined chemoradiation further delayed growth relative to either modality alone in three of six PDX models. Following irradiation, two of three PDX models demonstrated sustained up-regulation of Hh transcripts. Combined LDE225 and radiation, and 5E1 alone delayed growth relative to either treatment alone in a Hh-responsive PDX model, but not in a non-responsive model.

**Conclusion:**

Hh signaling mediates the radiation response in some EAC PDX models, and inhibition of this pathway may augment the efficacy of radiation in tumours that are Hh dependent.

## Introduction

The incidence of esophageal adenocarcinoma (EAC) is rapidly rising, surpassing that of esophageal squamous cell carcinoma (ESCC) in the United States[1,2]. Chemoradiotherapy with or without surgery is one standard of care for patients with locally advanced disease[3], yet five-year survival remains ≤20% due to disease recurrence and metastasis after therapy[4]. Increasing radiation dose does not improve efficacy, and is associated with higher normal tissue toxicity and patient mortality[5]. Targeting pathways involved in radiation resistance is a potential method to improve outcomes.

The Hedgehog (Hh) pathway, a member of the stem cell signaling network, may contribute to radiation resistance in aerodigestive cancers. Binding of Hh ligands Sonic (SHH), Indian (IHH) or Desert Hedgehog (DHH) to the transmembrane receptor Patched-1 (PTCH1) removes PTCH1 repression of Smoothened (SMO), another transmembrane protein. SMO release causes the dissociation of a cytoplasmic inhibitory complex that, when assembled, targets the glioma-associated oncogene homologue (GLI) family of transcription factors, GLI1, GLI2 and GLI3 for proteolytic cleavage. With dissociation of this complex, GLI proteins accumulate and translocate to the nucleus[6]. Vertebrates have a second receptor isoform, PTCH2. Hh signaling regulates stem and progenitor cell proliferation and differentiation, tissue polarity, and is critical to the development of the esophagus[6,7]. In adult life, Hh signaling mediates tissue homeostasis and repair after injury[8–12]. The pathway is aberrantly activated in EAC and its precursor lesion, Barrett’s Esophagus (BE), and has been shown to promote columnar cell differentiation in the squamous lining of the esophagus after exposure to acid and bile salts [13–17]. A clinical SMO inhibitor prevented the development of BE and EAC in an *in vivo* model of gastroesophageal reflux[18]. Thus, reactivation of an embryonic pathway in response to tissue injury and inflammation may contribute to esophageal carcinogenesis[13,19,20]. It is unclear whether this phenomenon reflects on the adaptation of an epithelial cell to profound Hh dependency during inflammation, or represents the signaling mechanism of the tumour initiating cells (TIC) compartment[21–26]. Hedgehog inhibitors have anti-proliferative and pro-apoptotic effects on EAC *in vitro*[14,27], however *in vivo* evaluation has not been reported in EAC.

Hh signaling may mediate tissue response to injury from radiation. Pathway expression correlates with poorer patient outcomes following radiation/chemoradiation in several tumour sites [28–30], including EAC; recently, nuclear GLI1 staining of EAC specimens from patients treated with chemoradiation was shown to predict a lower probability of pathologic complete response.[31] Direct evidence of Hh signaling mediating radiation resistance in EAC is lacking. One *in vivo* study demonstrating increased Hh activity after chemoradiotherapy was subsequently shown to involve a contaminated non-EAC cell line[32,33]. The present study utilizes patient derived xenograft (PDX) models to interrogate Hh signaling as a radioresistance mechanism in EAC.

## Materials and methods

### Patient-derived xenografts (PDX)

Development, engraftment, gene expression profiles and chemosensitivities of our PDX models have been described previously[34,35]. Briefly, NOD/SCID and NOD/SCID/IL2Rγ^-/-^ were bred internally at the Ontario Cancer Institute Animal Care Facility and ranged in age from 4–6 weeks. Animals were treated according to the ethical guidelines of the University Health Network’s Animal Care Committee. Approval for xenograft experiments was granted by the University Health Network Research Ethics Board (UHN REB) (REB #06-0779-T). Animals were maintained in a pathogen-free environment and fed a sterilized pellet diet and water *ad libitum*. Euthanasia was performed by cervical dislocation or by inhaled nitrous oxide.

**Fig 1:**
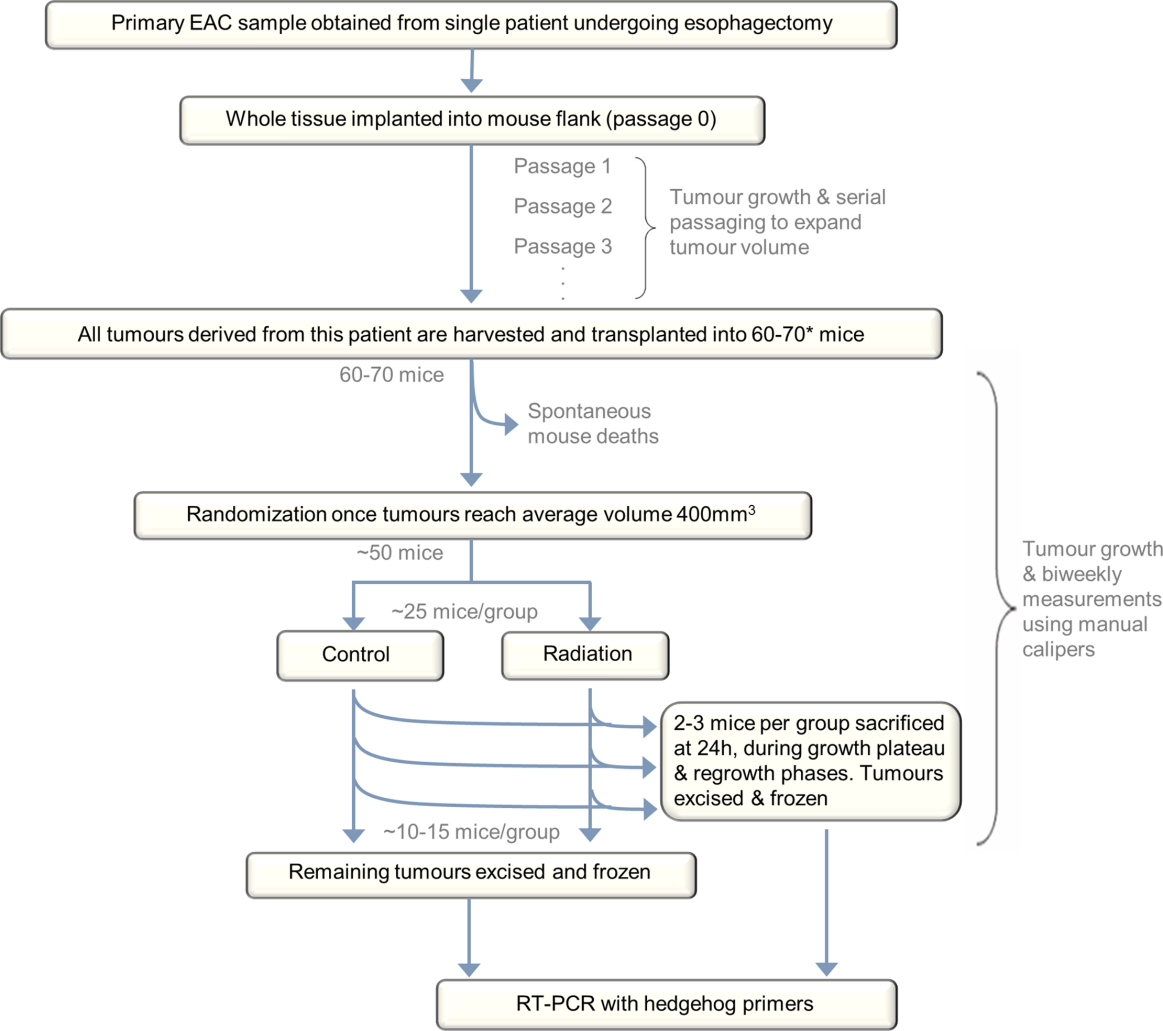
Xenograft experimental design. A radiation experiment is shown as an example. Similar protocols were used for chemoradiation and for hedgehog inhibitor experiments, albeit with larger numbers of mice and without RT-PCR. *Up to 90 mice were used for large hedgehog inhibitor experiments to ensure sufficient numbers remained at the conclusion of the experiment.

Primary EAC samples were obtained from consenting patients through the University Health Network Tissue Bank under Research Ethics Board approval for both tissue acquisition and animal use (REB #06-0779-T). All PDXs derived from one patient tumour are termed a PDX model. The primary patient tumour implanted into the first generation of mice is labeled passage 0, and transplantations into subsequent generations of mice are labeled in increasing order (Fig 1).

### Radiation and chemoradiation

Radiation and chemoradiation growth delay experiments were performed on seven and six PDX models, respectively, in order to select potentially useful models for Hh inhibition. PDX tumours were measured biweekly using calipers, and tumour size was calculated as *volume = length*×*width*^*2*^×*0*.*52* (ellipsoid). When tumour volumes reached approximately 400mm^3^, mice were randomized into control (non-irradiated) and irradiated groups for radiation growth delay experiments, and control, chemotherapy, radiation and chemoradiation groups for chemoradiation growth delay experiments (n ≥ 10 mice per group) (Fig 1). Tumour measurements were performed by an individual blinded to treatment arm. Irradiation occurred in the Spatio-temporal Targeting and Amplification of Radiation Response (STTARR) facility in Toronto, using equipment designed for animal models. 4Gy (3.07Gy/min) of X-rays was delivered using an XRAD 225 kVp precision irradiator fitted with a 2mm thick copper filter and 2.5cm diameter collimator centered on the tumour. Mice were restrained in a plastic container with the tumour-bearing leg extended from the body and secured in abduction. Cisplatin 5.4 mg/kg body weight (Hospira, DIN:02126613) and paclitaxel 9mg/kg (Hospira, DIN:02296624) were administered by intraperitoneal (i.p.) injection approximately one hour prior to irradiation. The control group received an i.p. injection of saline at an equivalent volume. Intraperitoneal injection is a well-accepted method of drug administration in murine models for the chemotherapy agents listed, due to superior technical feasibility compared with intravenous routes. This combination regime was selected based on initial chemosensitivity testing of PDX models[35].

### Evaluation of Hedgehog pathway activity

Initially, we attempted to evaluate the activity of the Hh pathway using both gene expression and protein expression. However, all commercially available Hh antibodies showed non-specific binding in both immunohistochemical staining and western blots of our PDXs to the extent that assessing protein expression was abandoned. This non-specific binding of antibodies to GLI1/2, PTCH1, SHH and SMO across all samples was consistent with those reported by other groups[6].

For gene expression, primers were designed for mouse and human *SHH*, *IHH*, *PTCH1*, *PTCH2*, *GLI1*, *SMO*, and the housekeeping genes *ACTB*, *HSP90AB1* and *YWHAZ* (S1 Table). Separate human and mouse-specific primers were desirable in order to differentiate transcriptional changes in the human tumour epithelium versus murine stroma. Housekeeping genes were chosen for expression stability after irradiation. The species specificity of each primer was tested by qRT-PCR on normal mouse liver and the human EAC cell line, OE33. Primers that cross-amplified in both species, or that produced doublet dissociation curves were redesigned. Two to three xenograft mice from each treatment group were sacrificed at multiple time points, including 24 hours after irradiation, during the plateau phase of the curve following treatment, and during the re-growth phase. Volume- or time-matched control tumours were sacrificed for comparison, depending on the experiment. Biopsied normal esophagus served as a control to determine baseline Hh expression in untreated EAC tumours. PDX tumours were immediately excised and frozen at -80°C in Optimized Cutting Temperature compound (Sakura Finetek). RNA was isolated from frozen whole tissue sections, and additional sections stained with hematoxylin and eosin were assessed by a pathologist. RNA was extracted using the RNeasy Mini Kit (Qiagen) with on-column DNA digestion, quantified using a Nanodrop spectrophotometer, and evaluated for quality using the Agilent 2100 Bioanalyzer. qRT-PCR experiments used RNA samples with RNA integrity numbers above 8.

RNA samples were reverse-transcribed (iScript™ cDNA Synthesis Kit (Bio Rad); RT^2^ SYBR® Green ROX™ qPCR Mastermix (Qiagen); ABI 7900HT (Life Technologies)) in triplicate wells, and quantified by qRT-PCR (Sequence Detection System v2.3). For each sample, a ΔC_t_ was calculated by normalizing to the geometric mean of the three housekeeping genes. ΔΔC_t_ was the difference between the ΔC_t_ of each irradiated sample and its matched control; fold differences were calculated as 2^-ΔΔCt^.

### Hh inhibition

Two PDX models with different responses in Hh gene transcription following irradiation were selected for combined Hh inhibitor-radiation experiments. PDX mice were randomized to five groups: (1) non-treated control, (2) inhibitor control, (3) inhibitor, (4) radiation and (5) inhibitor plus radiation. 5E1, purified from hybridoma supernatant obtained from the laboratory of Thomas Jessell, was given by i.p. injection (20mg/kg) 24 hours prior to irradiation. The inhibitor control was i.p. polyclonal mouse IgG antibody (20mg/kg). LDE225 (Novartis), a small molecule SMO inhibitor, was given by daily oral gavage (60mg/kg) for 21 consecutive days, beginning 24 hours prior to irradiation. The inhibitor control (“vehicle”) was a solution of 0.5%methycellulose/0.5%tween80 delivered by oral gavage (60mg/kg).

### Statistical analysis

Statistical analyses were performed by a biostatistician external to the original experiment. SAS9.3 and R were used. Two mathematical models, a two-slope mixed-effect repeated measures model and a linear mixed effect repeated measures model (both with random effects components) were used to describe PDX tumour growth, chosen based on best model fit (S1 Fig and S2 Table). These models generated a time for PDX growth to reach either twice the initial volume (*2V*_*0*_) or three-times the volume (*3V*_*0*_); *2V*_*0*_ was utilized when irradiation occurred at an average tumour volume >500mm^3^ (i.e. “late irradiation”). Growth delay was the difference in 2V_0_/*3V*_*0*_ between treatment and control groups, measured in days. To test for a trend in growth delay across passages (a “passage effect”), growth delays were first normalized to the intrinsic growth rate of each PDX model, and tested using linear regression; slopes with equivalency of zero across passages denoted a lack of a passage effect (*H*_*0*_: *λ*_*passage*_ = 0; see S3 Table). A chi-square test with one degree of freedom was used to test significance of absolute growth delays among treatment groups within one passage (*H*_*0*_: growth delay between two groups = 0).

## Results

### PDX models recapitulate the variable radiation/chemoradiation sensitivities seen clinically

**Fig 2:**
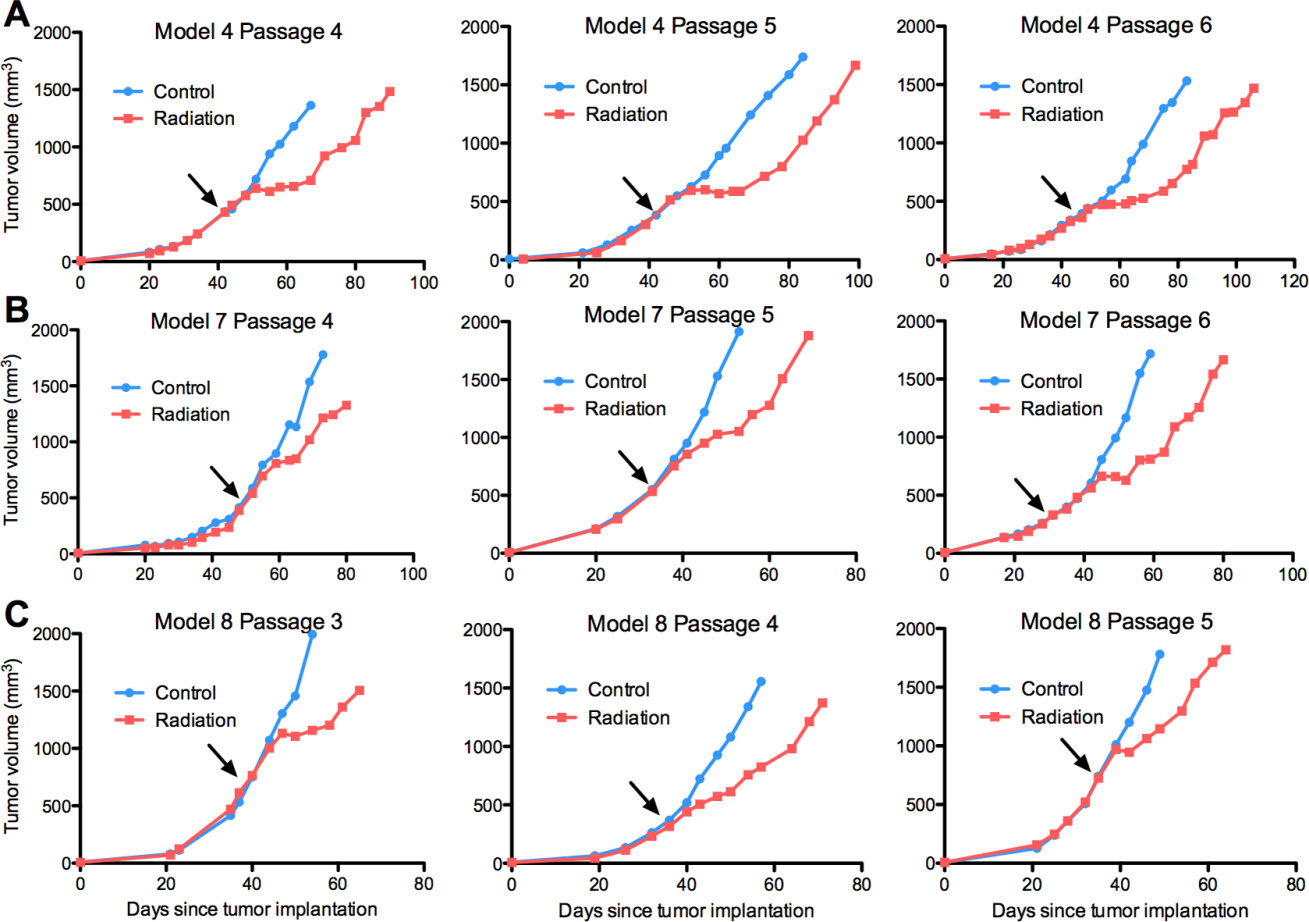
Radiation significantly delays PDX tumour growth across multiple passages. Three representative PDX models (rows **A**-**C**) are shown. Arrows indicate time of irradiation.

We first evaluated our PDX models using radiation and chemoradiation, current standard therapies for EAC. Seven PDX models were irradiated, six of which were treated on multiple passages. Radiation significantly delayed tumour growth in all seven models, measured by a change in slope relative to control tumours. There was no difference in specific growth delays (SGDs) across passages (S3 Table). Representative growth curves from three models irradiated over multiple passages are shown in Fig 2.

**Fig 3:**
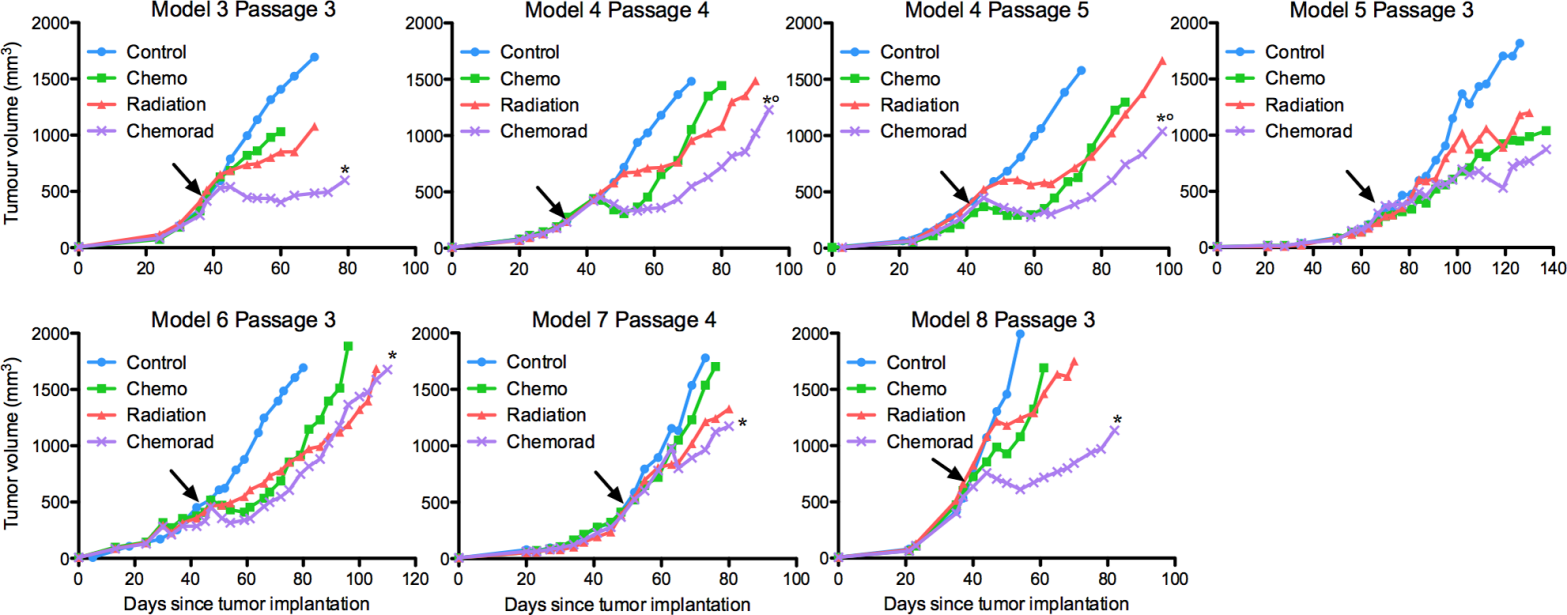
Chemoradiation growth delays vary across PDX models. Arrows indicate time of irradiation. Growth delays that are significant versus chemotherapy alone and versus radiation alone are marked with an asterisk and circle, respectively.

Combined chemoradiation significantly delayed tumour growth relative to one or both modalities alone in all PDX models with the exception of model 5, which showed a non-significant trend. Chemoradiation in model 4 significantly delayed growth relative to both single modality arms (p<0.05). The magnitudes of statistically significant chemoradiation growth delays ranged from 6–19 days versus radiation alone, and 6–37 days versus chemotherapy alone (p<0.05) (Fig 3, S4 Table).

### SHH transcription is higher in untreated EAC tumours versus normal esophageal epithelium

**Fig 4:**
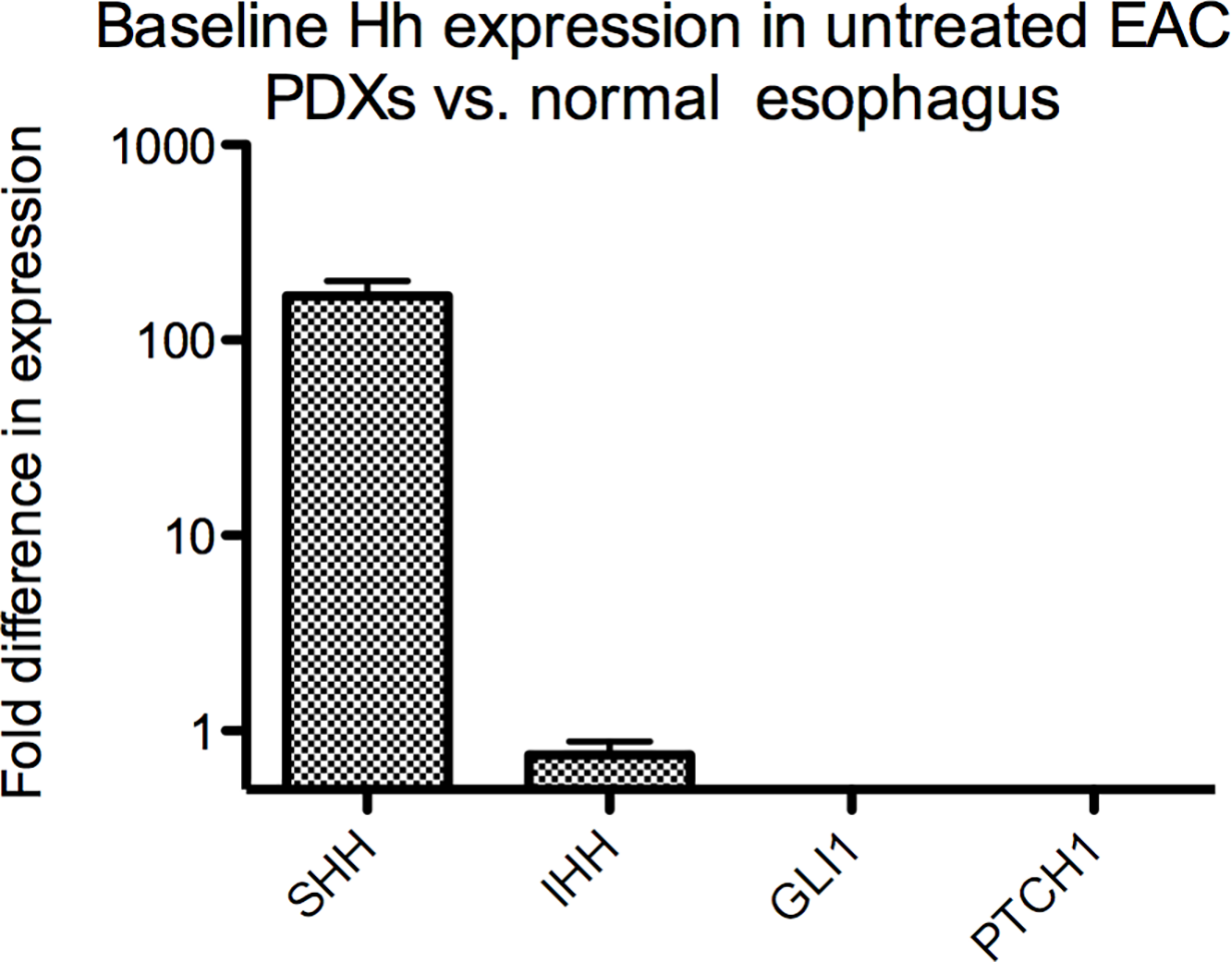
Baseline Hh expression in untreated EAC tumours versus normal esophagus using RT-PCR. Error bars represent standard error of the fold change. SHH was expressed 168-fold higher in untreated tumours. Fold changes in expression of IHH, GLI1 and PTCH1 were 0.75, 0.12 and 0.23, respectively.

We next assessed baseline Hh pathway activation by comparing Hh gene expression in untreated tumours (PDX model 8) to normal human esophagus. *SHH* was 168-fold up-regulated in untreated EAC compared to normal esophagus; however expression of *IHH*, *GLI1* and *PTCH1* was either equivalent or lower in EAC tissue *versus* normal esophagus (Fig 4).

### Hh pathway transcripts are present in both epithelial and stromal compartments

**Fig 5:**
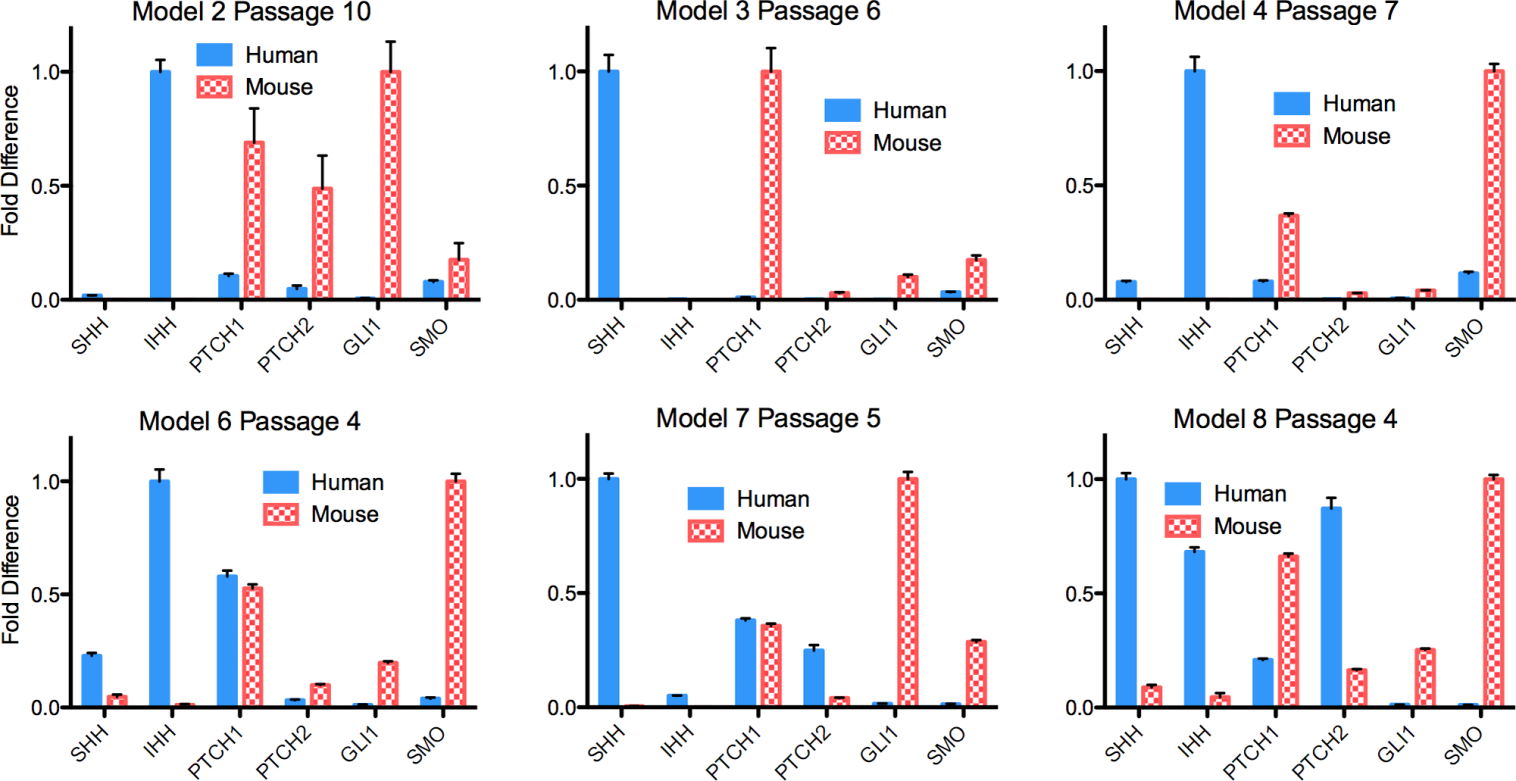
Baseline expression of Hedgehog transcripts in untreated EAC tumours from six PDX models. “Human” represents the patient-derived epithelium. “Mouse” represents the host stroma. Transcript levels are normalized to the highest expressed gene per species per model.

In an exploratory fashion, we compared the various Hh signalling pathway components by human and mouse-specific gene expression. Histologic examination confirmed the presence of both epithelium and stroma in all samples used for qRT-PCR, with epithelium ranging from 60–90%. *GLI1*, *PTCH1*, *PTCH2* and *SMO* were used as surrogate markers of Hh signalling activity[36]. In untreated PDX tumours, *SHH* and *IHH* were transcribed predominantly in the human-derived tumour epithelium, while *Gli1*, *Ptch1* and *Smo* were transcribed predominantly in the murine stroma (Fig 5). Although this might suggest an epithelial-to-mesenchymal paracrine mechanism at baseline, no firm conclusions can be made. That pathway receptors (*PTCH1* and/or *PTCH2*) were expressed at low levels in human epithelium from models 2 and 4, and at intermediate levels in the epithelium of models 6, 7, and 8 suggests a possible secondary autocrine signal.

### Radiation up-regulates Hh signaling in 2 out of 3 PDX models

We next asked whether precision irradiation induces Hh gene expression changes. Models 6, 7 and 8 were chosen for pragmatic reasons: resuscitation of other previously frozen models failed, and several concurrently available models were exhibiting features of late-passaging, including cystic growth or slow growth, rendering them infeasible for further evaluation.

**Fig 6:**
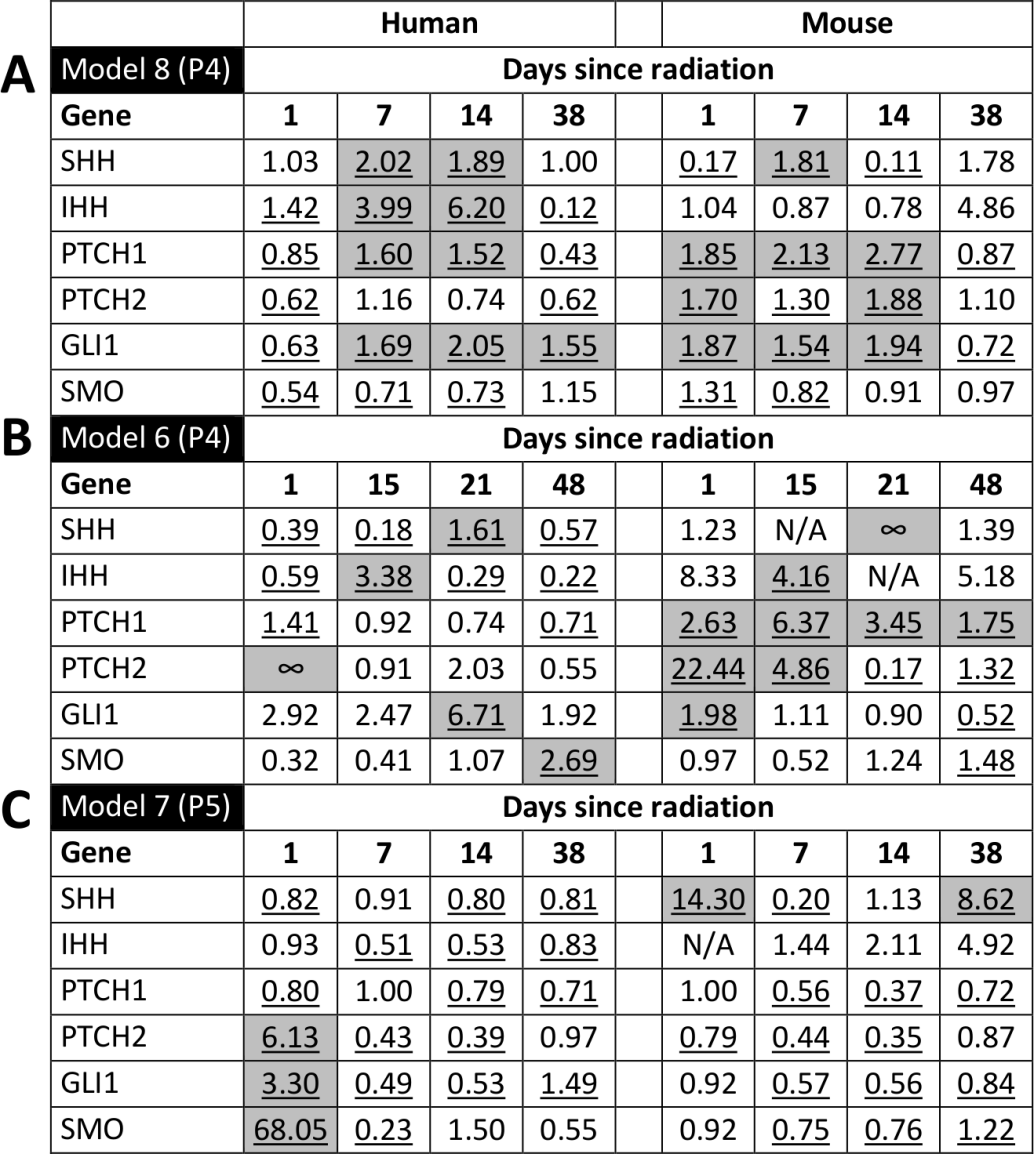
Radiation up-regulates Hedgehog transcription in some EAC tumours relative to controls. Significant (p<0.05) fold changes are underlined. Fold changes that are both ≥1.5 and statistically significant are shaded in grey. A lemniscate indicates that transcripts were undetectable in controls but detectable in treated tumours (infinite fold change). N/A indicates undetectable transcripts in treated tumours. (A-C) Fold changes in transcript levels in models 8, 6 and 7.

Models 6 and 8 showed similar pathway responses that were distinct from model 7 (Fig 6A–6C). In models 6 and 8, *SHH* and *IHH* were between 1.6- and 6.2-fold up-regulated in human tumour epithelium at one to three weeks following irradiation (p<0.05). In contrast, *Ptch1*, *Ptch2* and *Gli1* were between 1.5- and 22.4-fold up-regulated in murine stroma at 1–4 weeks following irradiation (p<0.05). Model 7 exhibited a unique expression profile that suggested a lack of sustained Hh response or Hh signalling independence after irradiation. Hh transcripts transiently increased in both epithelium and stroma immediately following radiation, but returned to baseline quickly, and remained mostly unchanged or down-regulated for the remainder of the experiment.

### SHH inhibition radiosensitized a Hh-expressing PDX model, but not a Hh-independent PDX model

If Hh up-regulation represents a potential mechanism of response to radiation, then pathway inhibition might prolong the radiation-induced growth delay of PDX tumours. Two PDX models, each with different Hh pathway responses to radiation were selected for inhibitor experiments. Models 7 and 8 were treated with 5E1, a monoclonal antibody for SHH. We hypothesized that co-treatment with 5E1 and precision irradiation would augment the growth delay of PDX tumours relative to either treatment alone in model 8 but not in model 7.

**Fig 7:**
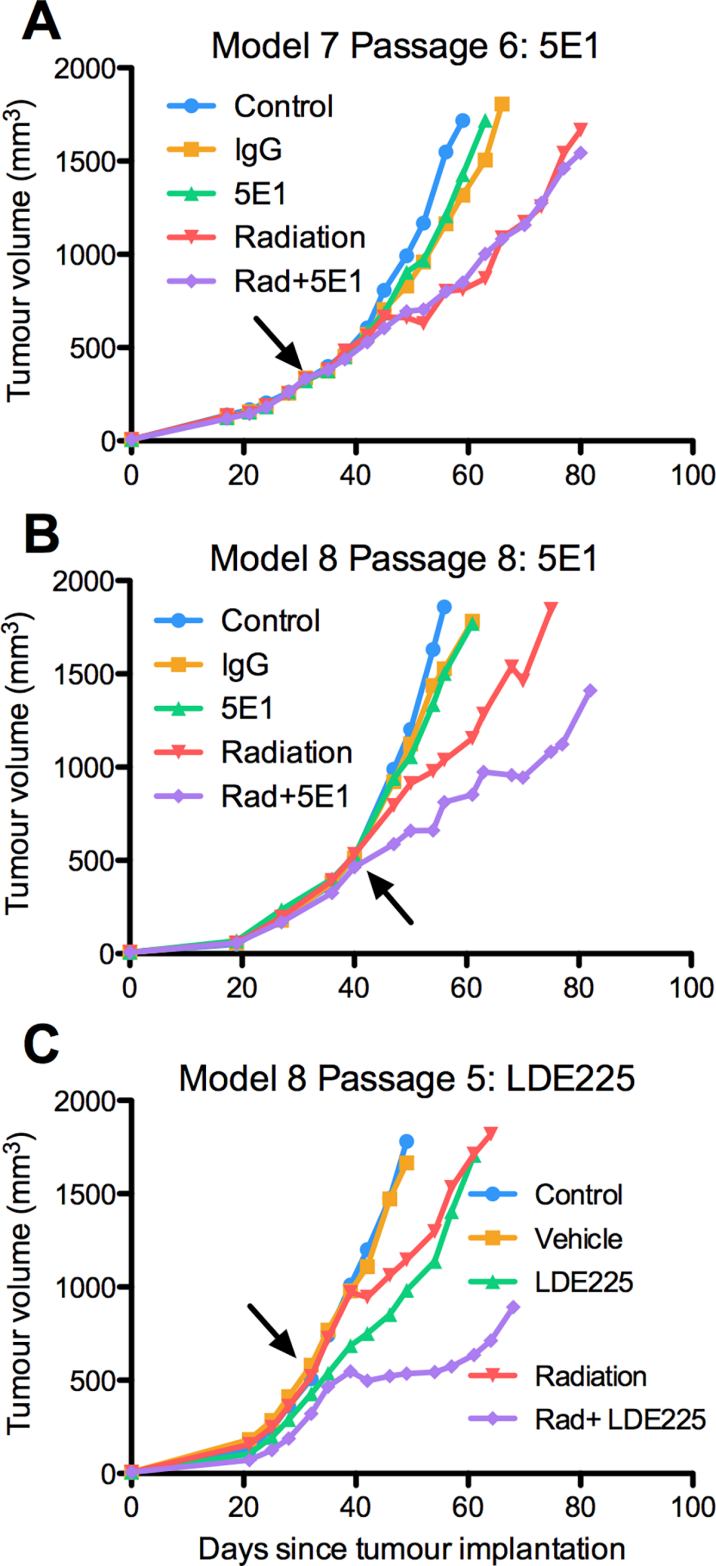
Hedgehog inhibition increases growth delay in some PDX models. Arrows indicate day of irradiation. **(A)** 5E1 used on a Hh-nonresponsive model. **(B)** 5E1 used on a Hh-responsive model. **(C)** LDE225 used on the same Hh-responsive model.

Radiation alone induced significant growth delays of 19 and 26 days in model 7 and model 8, respectively (p<0.001) (Fig 7A and 7B). In model 7, 5E1 alone did not significantly increase the growth delay relative to IgG (p = 0.2) or untreated control (p = 0.4), and 5E1 in combination with radiation did not significantly increase the growth delay relative to radiation alone (p = 0.8) (Fig 7A). In contrast, in model 8, 5E1 alone caused a small albeit significant growth delay of approximately 5 days relative to control (p = 0.02) and a non-significant growth delay relative to IgG (p = 0.1). Co-administration of 5E1 and radiation in model 8 caused a 7-day growth delay relative to radiation alone, however this effect was not significant (p = 0.2) (Fig 7B). Hh pathway inhibition was confirmed with RT-PCR of mouse GLI1 transcripts based on a paracrine signaling mechanism. In all three experiments (Fig 7), administration of 5E1 or LDE225, whether alone or in combination with radiation, resulted in a significant 0.01- to 0.38-fold change in mouse GLI1 transcript levels relative to IgG and “vehicle,” respectively.

### SMO inhibition radiosensitized the same Hh-expressing PDX model

We tested the same hypothesis using a clinically available SMO inhibitor, LDE225. By this time, model 7 failed to re-expand into a new cohort of mice, and the remaining experiment was performed solely on the radiation-associated Hh-dependent model 8. LDE225 alone caused a non-significant growth delay of approximately 2 days relative to untreated controls and vehicle-treated tumours in model 8 (p = 0.28 and p = 0.47, respectively) (Fig 7C). LDE225 administered 24 hours prior to and for three weeks following irradiation increased growth delay relative to both radiation and LDE225 alone. However, only the latter growth delay was significant (p = 0.16 and p = 0.004, respectively).

## Discussion

Data on whether chemoradiotherapy (CRT) is superior to radiation alone in EAC is limited, and while a small survival advantage has been demonstrated, neither modality is curative[37]. Our PDX models provide reasonable representations of EAC tumours, with variable sensitivities to radiation and chemotherapy, and an augmented response to CRT in some but not all models. Therapeutic responses were stably reproduced across multiple passages within the same model. Baseline transcription of SHH was higher in untreated EAC tumours compared to normal esophagus, mirroring previously published findings[27]. Two of three models up-regulated Hh transcripts following irradiation, indicating that the Hh pathway may contribute to the radiation response of some but not all tumours. Hh inhibition following irradiation delayed tumour repopulation in a model that was Hh-responsive but not in a Hh non-responsive model, suggesting a potential clinical role for inhibiting Hh signaling to augment radiosensitivity in a subset of EAC tumours.

Carcinogenesis is increasingly viewed as the misuse of homeostatic mechanisms involved in tissue repair and stem cell self-renewal, an appealing model for EAC as many cases develop from Barrett’s Esophagus via reflux-induced inflammation and injury within esophageal epithelium[38,39]. By extension, irradiation of EAC tumours may provoke the same stem cell-driven responses used during normal repair, including Hh activation. Indeed, Hh signaling has been shown to override cell cycle checkpoints and promote proliferation despite radiation-induced DNA damage[40–43].

Hh signaling in aerodigestive cancers has been described as autocrine, paracrine, and both[13–15,44]. Paracrine signaling in these tumours is thought to represent activation of the stromal compartment by ligand-producing epithelium. The ensuing structural and/or biochemical changes produce a tumour microenvironment favorable for tumour cell survival, growth and metastasis[45–47]. In our PDXs, up-regulation of Hh ligands was predominant in human epithelium, while up-regulation of GLI1 occurred in both compartments following irradiation. While this suggests both autocrine and paracrine signaling, non-canonical Hh signaling—which has been demonstrated in this cancer[48,49]—cannot be excluded here. Our data, though intriguingly obtained using human and mouse-specific primers, should not be used to decipher autocrine/paracrine mechanism, as our goal was simply to determine response of the Hh pathway to radiation. Quantitative *in vitro* evaluation at the protein level is necessary to further characterize these signaling patterns.

There are multiple ongoing or completed clinical trials involving Hh inhibitors alone or with standard therapy for aerodigestive cancers, including EAC [50–54]. The efficacy of Hh inhibitors may depend on their integration with other therapies, or on the selection of patients with Hh-dependent tumours [51]. In this study, we show that co-administration of radiation with the Hh inhibitors, 5E1 or LDE225, might improve outcomes in models where increased Hh signaling may contribute to the radiation response.

There are limitations to this study. Firstly, establishment of PDX models is imperfect and only a select subset engrafts successfully [34]. In addition, the effects of serially passaging these models can distort the original tumour features, weakening their advantage over cell lines. Because each experiment described herein required upwards of 90 mice, and because of issues preserving models over multiple passages, experimentation was limited to only a few PDX models. Further, while clinical management of EAC involves low dose-rate fractionated irradiation, we utilized single dose high dose-rate precision irradiation because of technical feasibility and our goal to interrogate gene expression changes immediately following irradiation. Future efforts to optimize the timing and dosing of pathway inhibitors in combination with radiation will require a clinically reflective model of fractionated irradiation.

Finally, our gene expression studies focused on the transcript level, since commercially available Hh antibodies showed non-specific binding in both immunohistochemical staining and western blots of our PDXs. Because of this, the use of separate murine and human primers must assume that both human SHH and murine Shh can elicit similar activation of the HH signaling in the recipient cells, regardless of the origin of species. While PDX responses to Hh inhibitors support to our gene expression data, only *in vitro* experiments using protein level read-out can conclusively implicate the pathway in this process.

## Conclusion

The Hh pathway is transcriptionally up-regulated following irradiation in two PDX models. One of these models had improved outcomes when Hh inhibition was added to radiation or chemoradiation. A third PDX model did not fit these expression patterns suggesting a lack of Hh-dependence, and pathway inhibition with 5E1 after conventional therapies failed to improve outcomes in this model. Thus, clinical integration of a SMO inhibitor after radiotherapy may improve patient response in a subset of EACs. Further efforts are needed to characterize this subset and to determine the timing of Hh inhibition relative to radiation or chemoradiation.

## Supporting information

S1 Fig Modeling of PDX growth curves. Representative curves of (A) the two-sloped mixed-effect repeated measures model and (B) the linear mixed effect repeated measures model. Models (red lines) were fitted to xenograft data (black circles) based on empiric evaluation of how well either model fit individual growth curves. Linear mixed effect repeated measures models were used for all control growth curves. The linear mixed effect model was as follows: log(*Volume*) = *β*_0_ + *β_Time_Time*+*β_Treatment_Treatment*+*β*_Time*Trteatment_Time*Trteatment+*α*_1,*T*_+*α*_2,*T*_*Time*.
Using this model, we tested the null hypothesis that radiation had no effect on tumour growth rate: *H*_0_: *β*_Time*Trteatment_ = 0.
(PDF)

S1 Table Primer sequences for RT-PCR. (PDF)

S2 Table Mathematical models used for each PDX growth curve. (PDF)

S3 Table Growth delay, specific growth delay and standard error for each PDX model and passage. A linear regression model was fitted to the specific growth delays for all passages within one PDX model to determine whether there is an effect of passage number on SGD. The mathematical model is given by *SGD* = *λ*_0_ + *λ_Passage_Passage*. The p-values in the right-hand column reflect the test for effect of increasing passage on SGD within each model. (i.e. “passage effect”).
(PDF)

S4 Table P-values for comparison of chemoradiation growth delay relative to either modality alone. (PDF)
