# Correction: Hedgehog inhibition mediates radiation sensitivity in mouse xenograft models of human esophageal adenocarcinoma

**DOI:** 10.1371/journal.pone.0224827

**Published:** 2019-10-30

**Authors:** Jennifer Teichman, Lorin Dodbiba, Henry Thai, Andrew Fleet, Trevor Morey, Lucy Liu, Madison McGregor, Dangxiao Cheng, Zhuo Chen, Gail Darling, Yonathan Brhane, Yuyao Song, Osvaldo Espin-Garcia, Wei Xu, Hala Girgis, Joerg Schwock, Helen MacKay, Robert Bristow, Laurie Ailles, Geoffrey Liu

The third, fourth, fifth, eleventh, twelfth, and thirteenth authors, Henry Thai, Andrew Fleet, Trevor Morey, Yonathan Brhane, Yuyao Song, and Osvaldo Espin-Garcia should not have been attributed equal contribution to this work. The last two authors, Laurie Ailles and Geoffrey Liu, should be noted as contributing equally to this work.
