# Supplementary material for: Hedgehog inhibition mediates radiation sensitivity in mouse xenograft models of human esophageal adenocarcinoma
Source: PLoS One. 2018 May 1;13(5):e0194809. doi: 10.1371/journal.pone.0194809 (PMC5929523; doi:10.1371/journal.pone.0194809)

S1 Fig. Modeling of PDX growth curves. Representative curves of (A) the two-sloped mixed-effect repeated measures model and (B) the linear mixed effect repeated measures model. Models (red lines) were fitted to xenograft data (black circles) based on empiric evaluation of how well either model fit individual growth curves. Linear mixed effect repeated measures models were used for all control growth curves.

The linear mixed effect model was as follows:

$$\log(Volume) = \beta_0 + \beta_{Time} Time + \beta_{Treatment} Treatment + \beta_{Time*Trteatment} Time * Trteatment + \alpha_{1,T} + \alpha_{2,T} Time$$

Using this model, we tested the null hypothesis that radiation had no effect on tumor growth rate: $H_0 : \beta_{Time*Trteatment} = 0$.

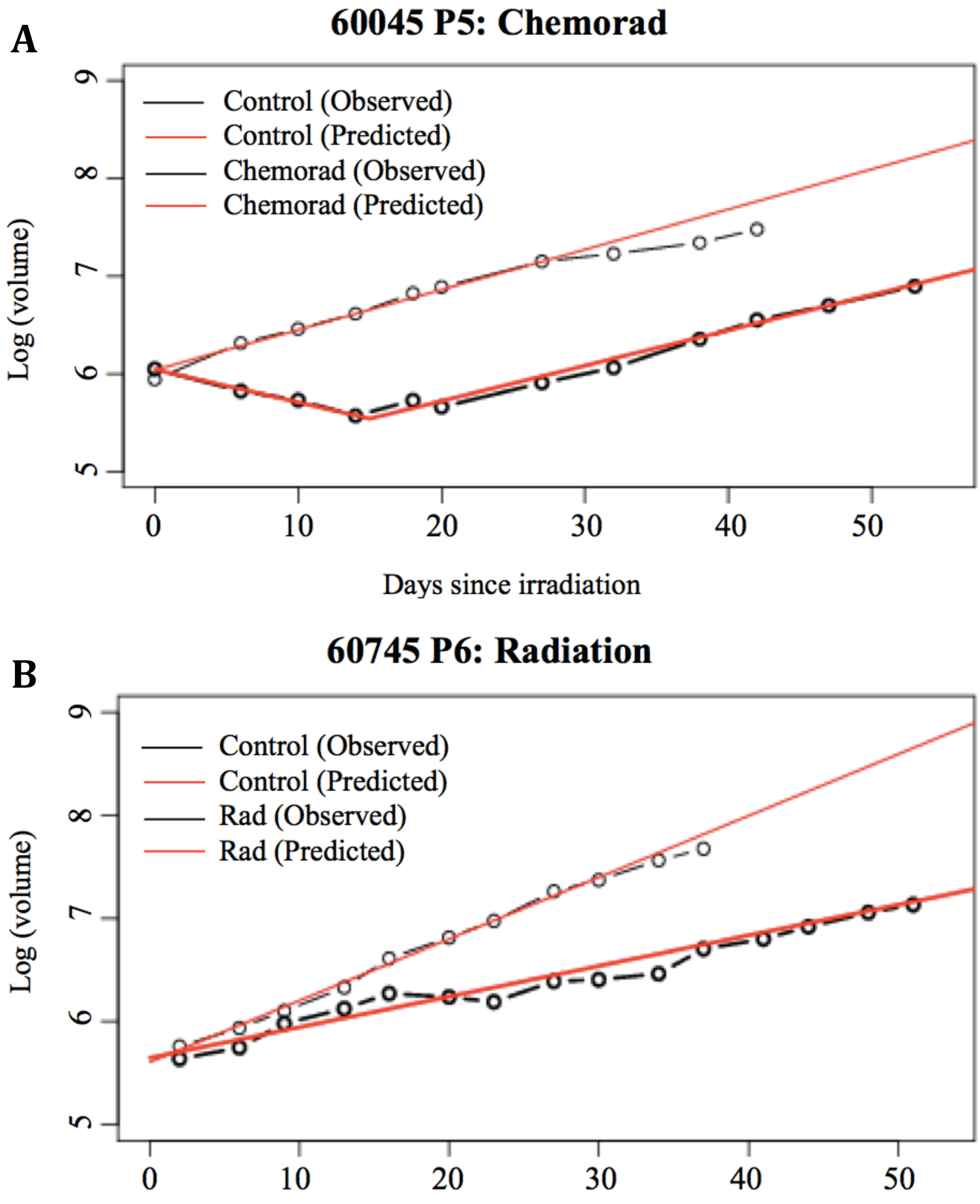

Supplement: S1 Fig — Representative curves of (A) the two-sloped mixed-effect repeated measures model and (B) the linear mixed effect repeated measures model. Models (red lines) were fitted to xenograft data (black circles) based on empiric evaluation of how well either model fit individual growth curves. Linear mixed effect repeated measures models were used for all control growth curves. The linear mixed effect model was as follows: log(Volume) = β0 + βTimeTime+βTreatmentTreatment+βTime*TrteatmentTime*Trteatment+α1,T+α2,TTime. Using this model, we tested the null hypothesis that radiation had no effect on tumour growth rate: H0: βTime*Trteatment = 0. (PDF) [file pone.0194809.s001.pdf]
