# Supplementary material for: Hedgehog inhibition mediates radiation sensitivity in mouse xenograft models of human esophageal adenocarcinoma
Source: PLoS One. 2018 May 1;13(5):e0194809. doi: 10.1371/journal.pone.0194809 (PMC5929523; doi:10.1371/journal.pone.0194809)
Supplement: S1 Table — (PDF) [file pone.0194809.s002.pdf]

Table S1: Primer sequences used for RT-PCR

Transcript	Human	Mouse
SHH	F: cagaggagtctctgcactacga<br>R: cgtagtacacccagtcgaagc	F: cactatgagggtcgagcagtg<br>R: gtggatgtgagctttggattc
IHH	F: acaaagcatgggacactggt<br>R: catgccaagctgtgaaagagt	F: gcattgctctgtcaagtctga<br>R: tctcctggctttacagctgac
PTCH1	F: caccgacacacacgacaatac<br>R: gcatggtaatctgcgtttcat	F: ctgctgggtgtactgatgctt<br>R: agcagaaccagtccattgaga
PTCH2	F: tcttctacatggggctgacc<br>R: gtatttgtcgtgcagccattc	F: ctccgctcaggtcattcagat<br>R: ggaggcaaaatggtgactaca
GLI	F: acacatatggacctggctttg<br>R: ctgccctatgtgaagccctat	F: tcctctcattccacaggacag<br>R: ctggtatgggagttcctggtt
SMO	F: tgagtgggatttgttttgtgg<br>R: ctcctcggatgaggaagtagc	F: agattgtttgccgagcagat<br>R: ccacgaaccagactactccag
ACTB	F: ttgctatccaggctgtgctat<br>R: agggcatacccctcgtagat	F: cgttgacatccgtaaagacctc<br>R: gtgctaggagccagagcagta
HSP90AB1	F: gtcttctgctggaggttcctt<br>R: ctttgacccgcctctcttcta	F: gaggcagacaaaaacgacaaa<br>R: tgagaaaccagaggagagcag
YWHAZ	F: aatgcttcacaagcagagagc<br>R: tgcttgttgtgactgatcgac	F: ctgcctacatattggtgtgtg<br>R: tttgtgtcacagcctcacaag
