# Supplementary material for: Hedgehog inhibition mediates radiation sensitivity in mouse xenograft models of human esophageal adenocarcinoma
Source: PLoS One. 2018 May 1;13(5):e0194809. doi: 10.1371/journal.pone.0194809 (PMC5929523; doi:10.1371/journal.pone.0194809)
Supplement: S2 Table — (PDF) [file pone.0194809.s003.pdf]

S2 Table. Mathematical models used for each PDX growth curve

Two-slope mixed effect repeated measures	Linear mixed effect repeated measures
Model 3 passage 3 (radiation, chemorad)<br>Model 4 passage 4 (radiation)<br>Model 4 passage 5 (radiation, chemo, chemorad)<br>Model 5 passage 3 (radiation)<br>Model 6 passage 3 (chemo, chemorad)<br>Model 8 passage 3 (chemorad)<br>Model 8 passage 5 (LDE225+radiation)	All other treatment groups<br>All control groups
