# Supplementary material for: Hedgehog inhibition mediates radiation sensitivity in mouse xenograft models of human esophageal adenocarcinoma
Source: PLoS One. 2018 May 1;13(5):e0194809. doi: 10.1371/journal.pone.0194809 (PMC5929523; doi:10.1371/journal.pone.0194809)
Supplement: S3 Table — A linear regression model was fitted to the specific growth delays for all passages within one PDX model to determine whether there is an effect of passage number on SGD. The mathematical model is given by SGD = λ0 + λPassagePassage. The p-values in the right-hand column reflect the test for effect of increasing passage on SGD within each model. (i.e. “passage effect”). (PDF) [file pone.0194809.s004.pdf]

S3 Table. Growth delay, specific growth delay and standard error for each PDX model and passage. A linear regression model was fitted to the specific growth delays for all passages within one PDX model to determine whether there is an effect of passage number on SGD. The mathematical model is given by $SGD = \lambda_0 + \lambda_{Passage} Passage$. The p-values in the right-hand column reflect the test for effect of increasing passage on SGD within each model. (i.e. "passage effect").

PDX Model	Passage	GD	SGD	SE_SGD	p-value for "passage effect"
2	8	7.47	0.47	0.19	0.261
2	10	18.70	1.14	0.43	
3	3a	26.21	1.21	0.31	0.402
3	3b	33.52	2.05	0.72	
3	4	34.67	1.10	0.92	
4	4	23.66	1.08	0.32	0.284
4	5	25.84	0.91	0.30	
4	6	20.76	0.75	0.18	
4	7	67.84	2.37	1.20	
5	3	16.48	0.56	0.28	NA (one passage only)
6	3	17.57	0.49	0.29	0.456
6	4	9.69	0.29	0.20	
7	4	11.15	0.56	0.13	0.373
7	5	13.67	0.73	0.13	
8	3	35.54	1.98	0.77	0.204
8	4	9.20	0.63	0.17	
