# Supplementary material for: Hedgehog inhibition mediates radiation sensitivity in mouse xenograft models of human esophageal adenocarcinoma
Source: PLoS One. 2018 May 1;13(5):e0194809. doi: 10.1371/journal.pone.0194809 (PMC5929523; doi:10.1371/journal.pone.0194809)
Supplement: S4 Table — (PDF) [file pone.0194809.s005.pdf]

S4 Table. P-values for comparison of chemoradiation growth delay relative to either modality alone.

PDX Model	Passage	Growth Delay Comparison	Growth delay using $3V_0$ or $2V_0$	GD (days)	P-value
3	3	Chemorad vs. Rad	$3V_0$	12	0.819
		Chemorad vs. Chemo		37	<0.001
4	4	Chemorad vs. Rad	$3V_0$	19	0.003
		Chemorad vs. Chemo		17	0.021
4	5	Chemorad vs. Rad	$3V_0$	6	0.013
		Chemorad vs. Chemo		25	<0.001
5	3	Chemorad vs. Rad	$3V_0$	44	0.134
		Chemorad vs. Chemo		39	0.194
6	3	Chemorad vs. Rad	$2V_0$	6	0.079
		Chemorad vs. Chemo		6	0.016
7	4	Chemorad vs. Rad	$3V_0$	6	0.362
		Chemorad vs. Chemo		17	<0.001
8	3	Chemorad vs. Rad	$2V_0$	10	0.166
		Chemorad vs. Chemo		30	<0.001
